# The Anatomy of the Human Ear, Illustrated by a Series of Engravings, of the Natural Size; with a Treatise on the Diseases of That Organ, the Causes of Deafness, and Their Proper Treatment

**Published:** 1817-08

**Authors:** 


*The Anatomy of the Human Ear, illustrated by a Series of Engravings, of the natural Size; with a Treatise on the Diseases of that Organ, the Causes of Deafness, and their proper Treatment.* By the late JOHN CUNNINGHAM SAUNDERS, Demonstrator of Anatomy, &c. &c. 1 vol. 8vo. pp. 112. Second Edition. London, 1817.

As the size and price of the first edition of this work greatly curtailed its circulation among the middling and inferior orders of the Profession, the present publishers could not do a more acceptable service to the classes in question, than thus to bring so invaluable a production within their reach. As the book ought now to be in every person's library, we shall not attempt any analysis, but merely give the Advertisement to this Edition.

" The very high estimation in which this work is held by the medical world, and by the anatomical student in particular, has induced the present publishers to bring forward a new edition in an octavo volume, that its usefulness may become more general from the portableness and convenience of its form, as well as on account of the reduced price at which it can be disposed. Another consideration has had much weight in determining the size, that of making it uniform with Mr. Saunders's work on the Eye; both of which, it is presumed, will long remain examples of the deep researches of a mind, whose wonderful penetration left no subject undeveloped to which he applied its powers, and which gained for its possessor a fame in the annals of science, which will only cease to exist when science itself shall fail to benefit mankind."—*Preface.*

We most strenuously recommend this little volume to the library of every member of the profession.

